# Bone Loss with Multiple Sclerosis: Effect of Glucocorticoid Use and Functional Status

**Published:** 2011-01-01

**Authors:** M H Dabbaghmanesh, Gh A Yousefipour

**Affiliations:** 1 Endocrine and Metabolism Research Center, Shiraz, Iran; 2 Department of Neurology, Nemazee Hospital, Shiraz University of Medical Sciences, Shiraz, Iran

**Keywords:** Multiple sclerosis, Bone mineral density, Ambulation, Steroid

## Abstract

**Background:**

Emerging data suggest a significantly increased prevalence of low bone mineral density (BMD) in men and women with multiple sclerosis (MS) compared to age matched controls. This study was performed to evaluate bone mineral mass in patients with MS in comparison to healthy age-and sex matched controls and to determine association of glucocorticoid use or ambulation ability with changing in bone mass in these individuals.

**Methods:**

Eighty two patients with MS and 328 age-sex matched healthy controls participated in the study. The Kurtzke expanded disability status scale (EDSS) was used to evaluate disability and functional capacity. Bone mineral density was measured using Dual X-ray absorptiometry. Serum calcium, phosphorus and 25(OH) vitamin D levels were assessed.

**Results:**

The MS patients had significantly lower BMD at the lumbar spines, neck and total femur compared to age-sex matched controls. EDSS scores were inversely correlated with total femur and spinal BMD. There was a negative correlation with cumulative steroid dose and BMD only for femoral neck.

**Conclusion:**

BMD was significantly lower in MS patients. Decreased ambulatory status and glucocorticoid usage were associated with low BMD in MS patients. These patients should be encouraged to increase mobility and to have protective measures to maintain bone mass.

## Introduction

Multiple sclerosis (MS) is a chronic inflammatory autoimmune disease of the central nervous system white matter resulting in extensive inflammation, demyelination and axonal damage. Classic features include motor weakness, paraparesis, paresthesia, loss of sight, diplopia, nystagmus, dysarthria, ataxia, impairment of deep sensation, and bladder dysfunction [1].

In vast majority of patients, the disease consists of alternating recurrent attacks followed by a period of a variable degree of recovery (relapsing remitting=RR) and sometimes of a steadily progressive one, especially in patients more than 40 years of age at the time of onset (primary progressive MS=PPMS), or as happens more often, an initially relapsing profile later becomes steadily progressive (secondary progressive MS=SPMS). Under the influence of corticosteroids, recovery from an acute attack, including an attack of optic neuritis, appears to be hastened. However, a substantial group of patients with acute exacerbations fail to respond. Although, there are some concerns for potentially harmful effects of steroids in long-term, results of studies in MS patients are controversial [2][3]. The other major causes of reduced bone mineral content in MS were estimated to be activation of immunoregulatory mechanism, poor ambulatory status, low exposure to sunlight and its consequences and vitamin D deficiency [4][5][6]. The aim of this study was to evaluate bone mineral density (BMD) in patients with MS in comparison to healthy age-sex matched controls and to determine the relationship of functional status and cumulative dose of corticosteroid usage with BMD in MS patients.

## Materials and Methods

The study was performed at Neurology Outpatients Clinic affiliated to Shiraz University of Medical Sciences from November 2006 to January 2008. Eligible patients had cerebral MRI abnormalities consistent with MS and clinically definitive MS [7]. Of the 216 patients who registered in this center; simple random sampling method was applied for selection of 90 cases in the range of 20-83 years old. All were accepted except 8 individuals. The source population for control group consisted of residents who lived in Shiraz, the Capital of Fars Province in southern Iran. The city map was divided into 40 areas. In each area, homes with postal code ended in zero were selected, and in each home, one adult person was enrolled. For each case, four control subjects were considered and were age-sex matched to the index cases. Eighty two cases (66 females, 16 males) and 328 eligible age-sex matched controls were invited to participate in this investigation. The individual were not included in the control group if they had a history of chronic medical problem, chronic use of medications such as thyroid hormone, vitamin and mineral supplements, anticonvulsants, bisphosphonate and hormone replacement therapy, calcitonin and selective estrogen receptor modulating agent which could affect bone metabolism, and also individuals who had undergone gastric surgery, those with special nutritional habits, pregnancy, and lactation during study. None of the patients had these exclusion criteria however the MS patients received calcium and vitamin D (500 mg calcium carbonate and 200 IU vitamin D respectively daily) as supplement. All participants were informed about the study and a written consent was obtained. Patients were interviewed by the same physician, regarding corticosteroid usage. Information were obtained from their charts and interviews. Cumulative IV cycles and oral daily steroids dosage were estimated as a sum of corticosteroids (prednisolon equivalent) used in milligram Patient disability status was assessed by assigning scores on the Kurtzke expanded disability status scale (EDSS), and this was done by the same neurologist [8]. We measured bone mineral density (BMD) with posteroanterior projection, using standard techniques from dual-energy X-ray absorptiometry (DPX-IQ, Lunar Co., Madison, WI, USA). The variation coefficient for consecutive determinations on spine and femur images in our laboratory was 1.2% at the lumbar spine and 1.1% at the femur region. Values for results of DXA assessments were expressed as BMD (g/Cm^2^) and Z scores of a healthy reference population, as supplied by the manufacturer. Blood were obtained after overnight fasting, and precautions were taken to avoid contamination. The sera were stored at -20 C until analysis was done. Serum chemical estimations were performed using Hitachi 902 autoanalyser (Boehringer Mannheim, Mannheim, Germany). Serum calcium (8.6-10.3 mg/dl), phosphorous (2.6-4.5 mg/dl), alkaline phospatase (<211 IU/L) and creatinin (0.6-1.1 mg/dl) were determined. The albumin corrected calcium was calculated according to the method of Payne et al [9]. Serum 25-OHD (23.1-113.0 nmol/l) level was assessed using immunoradiometric assay with the IDS gamma-B 25-hydroxyvitamin D kit (IDS, Fountain Hills, Arizona). Vitamin D deficiency was defined as a 25-hydroxyvitamin D level of less than 23.1 nmol/l.

Statistical analysis was performed using the statistical package for social sciences (SPSS, version 13.5, Chicago, IL, USA). Data were presented as mean (±SD) and Pearson correlation coefficients were used to assess relationships between parameters. Differences between patient and control group were assessed using the t-test. Multivariate linear regression analysis was used to estimate the independent effects of some variables on BMD.

Statistical significance was set at p<0.05. The study protocol was approved by Reviewer Board of Shiraz Endocrine Research Center and Shiraz University of Medical Sciences Ethics Committee.

## Results

Eighty two patients (66 females, 16 males) with mean age 36.1±8.9 years and 35.3±7.4 (p=0.76) respectively were enrolled in the study (p=0.762). The mean disease duration was 6.3±3.8 years in women and 7.8±5.2 years in men (p=0.209). Patient and control demographic data, biochemical profile and bone mass results are shown in Table 1. There was no significant difference in age, weight, height, menopausal age and body mass index between patients and controls. There was no significant difference in prevalence of menopausal state between case and control groups in women. (10.5% vs 9.3%).

**Table 1:** Demographic charectestics, bone mineral density assessment and biochemical profile in patients and controls.

	**Patients**	**Controls**	***P***
	**Mean (SD)**		
No. (M/F)	82 (16/66)	328 (64/264)	
Age (years)	35.9 (8.5)	36.2 (8.8)	0.804
Disease duration (years)	6.6 (4.1)		
Height (Cm)	164.9 (8.7)	164.3 (8.3)	0.550
Weight (Kg)	61.7 (13.1)	64.3 (12.8)	0.450
Body mass index (kg/m^2^)	22.6 (4.3)	24.6 (4.9)	0.561
Menopausal age	44.6 (3.7)	44.5 (6.5)	0.872
EDSS	2.6 (2.3)		
Cumulative steroid dosage (mg)	16038.5 (8878.7)		
Calcium (mg/dl)	9.3 (0.57)	9.3 (.67)	0.327
Phosphorus (mg/dl)	3.8 (0.61)	3.5 (0.66)	0.485
25(OH)D (nmol/l)	55.7 (54.2)	30.4 (21.5)	<0.001
Spine BMD (L2-L4)	1.08 (0.15)	1.13 (0.14)	0.015
Spine BMD (Z score)	-0.75 (1.2)	-0.48 (1.1)	0.038
Femur neck BMD	0.86 (0.19)	0.92 (0.13)	0.002
Femur neck (Z score)	-0.81 (1.4)	-0.37 (0.92)	0.002
Femur total BMD	0.91 (0.21)	0.96 (0.15)	0.034
Femur total (Z score)	-0.6 (1.6)	-0.18 (0.94)	0.015

Dividing the MS patients in RRMS and SPMS groups, 56% of patients were RRMS, 44% were SPMS. When we assessed these parameters between two groups, we found lower BMD in SPMS, however the only significant differences was the mean of cumulative steroid dosage which received and the mean of EDSS score (Table 2).

**Table 2:** Demographic charectestics, bone mineral density assessment and biochemical profile between RRMS and SPMS.

	**RRMS**	**SPMS**	***P***
Age (years)	35.3(10.1)	36.6(6.9)	0.544
Disease duration (years)	6.0(4.1)	7.2(4.0)	0.218
Height(Cm)	163.8(6.9)	167.1(10.9)	0.147
Weigh t(Kg)	62.9 (12.1)	62.1 (15.3)	0.808
Body mass index (kg/m^2^)	22.3(4.0)	22.7 (4.4)	0.456
EDSS	1.0 (1.3)	4.5 (1.2)	<0.001
Cumulative steroid dosage (mg)	11587(7626.2)	21151 (7055.6)	<0.001
Calcium (mg/dl)	9.5 (0.53)	9.2 (0.61)	0.075
Phosphorus (mg/dl)	3.8 (0.60)	3.8 (0.68)	0.937
25(OH)D (nmol/l)	45.3 (44.3)	65.5 (65.5)	0.190
Spine BMD (L2-L4)	1.11 (0.12)	1.07 (0.19)	0.262
Femur neck BMD	0.89 (0.16)	0.84 (0.24)	0.317
Femur total BMD	0.95(0.15)	0.89(0.28)	0.980

We observed negative correlation between BMD assessment in lumbar spines (r=-0.25, p=0.036), neck of femur (r=-0.26, p=0.027) and duration of disease in MS patients. Correlation between disease duration and cumulative steroid usage was statistically significant (r=0.25, p=0.036). 40.3% of MS patients had low serum vitamin D concentration. However we found lower prevalence of vitamin D deficiency in MS patients than controls (55.4%) whereas they received calcium and vitamin D supplementation. Vitamin D levels were correlated with BMD assessment at the lumbar spine (r=0.29, p=0.025). We did not find any correlation between vitamin D concentration and BMD assessment in proximal femur. Patients with MS had significantly a lower BMD at the lumbar spine, femoral neck and at total femur when compared to the age and sex matched individuals. BMD of the lumbar spines and femoral neck was significantly lower in these patients in comparison with normal subjects of the same age and sex (Z score) (Table 1). BMD values were found to be significantly lower in MS patients versus controls at lumbar spine and femoral sites in both genders (Table 3).

**Table 3:** Comparison of BMD between MS and control groups (mean±SD).

**Group**	**MS**	**Control**	***P***
Female			
Spine BMD (L2-L4)	1.09 (0.16)	1.13 (0.14)	0.018
Femur neck BMD	0.85 (0.19)	0.90 (0.13)	0.004
Femur total BMD	0.90 (0.22)	0.95 (0.14)	0.032
Male			
Spine BMD (L2-L4)	1 .05 (0.14)	1.12 (0.14)	0.012
Femur neck BMD	0.88 (0.17)	1.00 (0.14)	0.003
Femur total BMD	0.93 (0.16)	1.03 (0.14)	0.038

EDSS score were inversely correlated with BMD assessment at the lumbar spine (r=-0.37, p=0.002), femur neck (r=-0.38, p=0.002) and total femur (r=-0.34, p=0.005). There was a negative correlation with cumulative steroid dose and BMD only for femoral neck (r=-0.2, p=0.045). Analysis of the relationship between proximal femur, lumbar spines BMD and EDSS, using partial correlation coefficients controlled for age, BMI, and sex revealed a continuing negatively significant relationship with EDSS. Multiple regression analysis was considered to assess BMD in regard to the association with different variables (Vitamin D levels, cumulative steroid usage and EDSS status). Analysis revealed that EDSS was negatively related to BMD measurements at the lumbar spines (p=0.03), femoral neck (p=0.01) and total femur (p=0.038) whereas vitamin D was significantly related to BMD assessment only for lumbar spines (p=0.024) and the cumulative steroid dose was negatively related to femoral neck BMD (p=0.039).

## Discussion

Emerging data suggest a significantly increased prevalence of osteoporosis in men and women with MS compared to age matched controls. Other studies have shown that disability and corticosteroid use can decrease the BMD. This fact can be seen in both genders. Our findings showed a reduction of BMD compared with an age and sex-matched healthy population. Furthermore, we showed a significantly higher prevalence of low bone mass in MS patients than controls. We found lower BMD in MS patients with SPMS than the patient with RRMS, however this difference was not significant. Similar to other findings, we found that irreversible disability occurred more in patients with progressive disease than in those with a relapsing-remitting course and the patient with SPMS receiving higher cumulative dose of corticosteroid [10]. These parameters may explain the lower BMD in patients with MS and those with SPMS. Our results showed that there is a negative correlation between BMD values and duration of disease. The duration of disease was determined as the main variable affecting bone density in patients. Disability, glucocorticoid use in this duration and the common cytokines playing a role in the pathogenesis of both MS and osteoporosis might be responsible [11][12].

In this study, the average dosages of corticosteroid received by MS patients were inversely correlated with BMD assessment in femoral neck. Corticosteroids are widely used in medicine and they have saved lives, prevented blindness from the result of optic neuritis and revolutionized the management of autoimmune disease but they have brought their own complications and are considered as a risk factor for osteoporosis [13]. This is mainly due to corticosteroid action on cells called fibroblasts and osteoblasts which make the important collagen fibers and the microcrystals of bone material attached to them. Bone is particularly rich in collagen fibers but corticosteroids shut down the ability of fibroblast to make these fibers.

As a result, bones become less dense, more brittle and liable to fracture very easily [14]. Although corticosteroid treatment on skeletal and mineral metabolism have been assessed in MS patients, the results of studies are contradictory. Some studies showed glucocorticoid treatment in MS patients causing a decrease in bone formation, increase in bone resorption and there is a negative correlation between BMD and cumulative steroid dosage [15][16]. On the other hand, other studies did not find any data for glucocorticoid therapy to exert a negative impact on BMD in lumbar spine and femoral neck in patients with MS. So it suggests that decreased mobility may contribute to a decrease in bone mineral density more than corticosteroid use[3][17]. Furthermore, glucocorticoids have suppressive effects on cytokines that are involved in pathophysiology of MS and therefore it may have beneficial effects on patients wellbeing and mobility status [18].

EDSS scores were inversely correlated with BMD at the lumbar spine, femur neck and total femur. Bone loss occurred rapidly in relation to immobilization. The underlying mechanism has been attributed to weight-bearing changes at the proximal femur and spine, because most tetra/paraplegic patients bear minimal weight on these regions [19][20].

These results reinforce the findings from previous studies showing that disability with lower EDSS, the consequent immobilization, and corticosteroid use contribute to osteoporosis in both genders [12] So strengthening exercises for improving ambulatory status during rehabilitation programs and protective measures may have special importance in increasing spinal and proximal femur BMD in these patients. We found high prevalence of vitamin D deficiency in our patients in spite of receiving calcium and vitamin D supplementation. There is substantial evidence that vitamin D insufficiency increase the risk of falls and fractures [21]. Therefore active detection of vitamin D insufficiency among MS patients and intervention to restore vitamin D status to adequate concentration should be considered. Vitamin D repletion in MS patients who are deficient might reduce, to some extent, the rate of bone loss and decrease osteoporosis related fracture [5]. BMD is significantly lower in MS patients which may contribute to an increase in risk of fracture. The patient’s ambulatory status and glucocorticoid usage are determinative factors for osteoporosis in MS. Preventing vitamin D deficiency and active detection of vitamin D insufficiency among people with MS should be considered. These patients should be encouraged to increase mobility and to have protective measures to maintain bone mass.
